# Stronger Parental Than Temperature Effects on Methylation in Juvenile Brown Trout (*Salmo trutta*)

**DOI:** 10.1002/ece3.72154

**Published:** 2025-09-10

**Authors:** Shenglin Liu, Bror Jonsson, Larry Greenberg, Michael M. Hansen

**Affiliations:** ^1^ Department of Biology Aarhus University Aarhus C Denmark; ^2^ Norwegian Institute for Nature Research Oslo Norway; ^3^ River Ecology and Management Group, Department of Environmental and Life Sciences Karlstad University Karlstad Sweden

**Keywords:** climate change, epigenetics, methylation, phenotypic plasticity, transgenerational plasticity, whole‐genome bisulphite sequencing

## Abstract

Epigenetic modifications, particularly DNA methylation, are increasingly recognized as mechanisms underlying phenotypic plasticity and potential mediators of transgenerational responses to environmental change. We investigated the persistence of early life temperature‐induced DNA methylation changes and the role of parental life history in shaping methylation patterns in juvenile brown trout ( *Salmo trutta* ). Fertilized eggs from crosses of anadromous and resident trout were incubated under natural or elevated temperatures (by +3°C) until first feeding, after which all fish were reared under common conditions. Whole‐genome bisulfite pooled sequencing was conducted on juveniles 10.5 months post‐fertilization. We found weak and inconsistent evidence for persistent temperature‐induced methylation changes, with little overlap among different parental cross types. In contrast, parental life history, particularly maternal origin, significantly influenced offspring methylation patterns. Maternally derived differences were more extensive than paternal effects and were enriched for genes related to metabolism, nervous system function, and digestion, suggesting potential adaptive relevance. These findings suggest a limited long‐term impact of early‐life thermal conditions on methylation and emphasize a stronger role of transgenerational epigenetic effects in brown trout. Given that climate change is expected to alter thermal regimes in future aquatic ecosystems, our results, along with other recent publications, suggest that parental environmental history may be a more significant driver of epigenetic variability than the temperature experienced during early life. Understanding such mechanisms is critical for predicting how populations may respond to ongoing and future climate change.

## Graphical abstract

We investigated the persistence of early‐life temperature‐induced DNA methylation and the role of parental life history in shaping epigenetic patterns in juvenile brown trout. While early thermal conditions had limited long‐term effects, parental, especially maternal, life history exerted strong and functionally relevant influences on offspring methylation, suggesting a key role for transgenerational epigenetic inheritance under climate change.

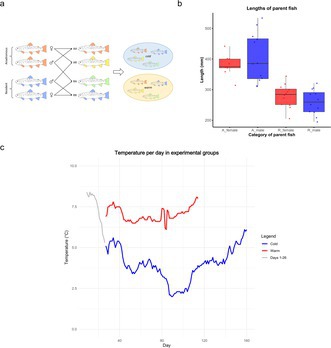

## 1 Introduction

The resilience and adaptability of populations and species to anthropogenic climate change represent some of the most pressing research questions in biology (Catullo et al. 2019; Hoffmann and Sgro 2011; Jørgensen et al. 2022; Wiens 2016). For instance, do biological traits evolve quickly enough and how does phenotypic plasticity interact among populations to mitigate the effect? The possible responses by organisms to climate change have typically been subdivided into (1) non‐genetic acclimation within a life span and (2) genetic adaptation through microevolution across generations (Hoffmann and Sgro 2011). Acclimation involves phenotypic plasticity, that is the ability of an organism to express different phenotypes in response to varying environmental factors (Pigliucci et al. 2006; Schlichting 1986). This is considered the most commonly observed type of response to climate change (Merila and Hendry 2014), although in reality phenotypic plasticity and microevolution may interact rather than being alternative types of responses (Catullo et al. 2019). The molecular underpinnings of phenotypic plasticity are complex, but it has been suggested that epigenetic factors play important roles (Angers et al. 2020; Ashe et al. 2021; Duncan et al. 2014; Schlichting and Wund 2014). It is therefore highly relevant to consider epigenetic mechanisms in the context of organisms' responses to the increasing temperature of climate change.

Epigenetics can be defined as modifications to the DNA that affect gene expression without changing the DNA sequence itself (Dupont et al. 2009), with methylation being by far the most studied mechanism (Anastasiadi, Venney, et al. 2021). This involves the addition of a methyl group to a nucleotide, in animals primarily Cytosine (C) at CpG sites (C followed by Guanin). Gain or loss of methylation in regulatory regions can regulate the expression of genes and in vertebrates, hypomethylation of promoter regions generally leads to increased transcription. Environmental inducibility of methylation processes and its ability to persist across cell divisions (Feil and Fraga 2012) suggests that methylation induced at juvenile life stages by environmental factors such as temperature can persist and modulate the phenotypes of the same individuals later in life. This means that methylation could be at least partly responsible for the phenomenon of developmental phenotypic plasticity (Jonsson et al. 2022; Nettle and Bateson 2015). If the environmental factors represent reliable cues predicting future environmental conditions, and if the induced phenotypic change affects fitness positively, then such developmental plasticity could be adaptively significant; but it could also be non‐ or even maladaptive, notably in unpredictable environments or if the induced phenotypic change itself does not increase fitness (Bateson et al. 2014).

In addition to the persistence of induced methylation changes within a lifespan, the possibility also exists for methylation to be transferred across generations. Transgenerational inheritance is well documented in plants and some invertebrates, whereas the situation is more complex in vertebrates (Anastasiadi, Venney, et al. 2021; Reik et al. 2001). In mammals, methylation erasure and reprogramming occur in primordial germ cells and in early embryonic stages, which should preclude transgenerational transfer of methylation (Guo et al. 2014; von Meyenn and Reik 2015; Wang et al. 2014). Nevertheless, recent results from mice suggest that induced methylation in promoter regions may be retained across generations, possibly involving some sort of DNA methylation memory outside the DNA itself (Takahashi et al. 2023). In fishes, methylation erasure and reprogramming vary across species (Kho and Ruzzante 2024). Hence, medaka ( *Oryzias latipes* ) shows patterns resembling those observed in mammals (Wang and Bhandari 2020), whereas in zebrafish ( *Danio rerio* ) methylation erasure and reprogramming are weaker or do not occur (Ortega‐Recalde et al. 2019; Skvortsova et al. 2019). In the latter species, it is primarily methylation in sperm that is the paternal contribution that is retained (Jiang et al. 2013), but there is also evidence of maternal transmission of methylation in, for example, chinook salmon ( *Oncorhynchus tshawytscha* ) (Venney et al. 2020). Regardless of the specific mechanisms, there is now evidence for parental transfer of methylation in a range of fish species such as brook charr ( *Salvelinus fontinalis* ), Atlantic salmon ( *Salmo salar* ), chinook salmon, and Atlantic cod ( *Gadus morhua* ) (Puvanendran et al. 2023; Venney et al. 2020, 2022; Wellband et al. 2021). Similar to phenotypic plasticity, environmentally induced methylation change in parents inherited by offspring (cross‐generational plasticity) could be adaptive if the environmental conditions experienced by parents are reliable cues for future environmental conditions experienced by offspring, and if it induces phenotypic change that increases fitness (Kappeler and Meaney 2010; Sheriff and Love 2013).

There are still relatively few examples of a direct link between methylation and phenotypic plasticity for specific traits (Ge et al. 2018; Heckwolf et al. 2020; Lindner et al. 2021; Morgan et al. 1999), but many studies have reported environmentally induced changes in methylation that persist into later life stages and likely have phenotypic effects (Anastasiadi, Shao, et al. 2021; Baerwald et al. 2016; Bentz et al. 2021; Bock et al. 2022; Le Luyer et al. 2017; Metzger and Schulte 2017; Todd et al. 2019). In ectotherms such as fishes that deposit their eggs and embryos at specific sites, temperature is one of the most important environmental factors affecting the development of the offspring. Several studies have shown that temperature induces methylation change (Anastasiadi, Shao, et al. 2021; Liu et al. 2022; Metzger and Schulte 2017; Puvanendran et al. 2023; Ryu et al. 2018; Savilammi et al. 2021; Venney et al. 2022), but less is known about the extent to which temperature‐induced methylation in early life persists into later stages. At the phenotypic level there is evidence for higher incubation temperature of eggs affecting physiological and life history traits later in life (Durtsche et al. 2021; Greenberg et al. 2023; Jonsson and Greenberg 2022), although it is not known if this has a basis in differential methylation. Anastasiadi, Shao, et al. (2021) studied European sea bass ( *Dicentrarchus labrax* ) exposed to a simulated marine heat wave few days after hatching, and found differentially methylated regions (DMRs) relative to a control group, and this difference persisted 3 years after the heat treatment. In contrast, another recent study of brook charr found negligible effects of temperatures experienced by juveniles on methylation later in life, and instead found a major parental influence, that is, transgenerational effects (Venney et al. 2022).

Here we focus on a salmonid fish species, the brown trout ( *Salmo trutta* ), which is expected to be substantially impacted by climate change (Jonsson and Jonsson 2009; Santiago et al. 2016). Spawning occurs in the autumn when fertilized eggs are deposited and incubated in gravel beds. The embryos experience the surrounding ambient water temperatures as they remain in place in the gravel, until they emerge as fry for first feeding in spring (Elliott 1994). Previous studies of brown trout have demonstrated phenotypic plasticity and locally adapted temperature reaction norms at early life history traits (Jensen et al. 2008) and at the level of transcriptomes (Meier et al. 2014).

Many populations of brown trout show partial migration and facultative anadromy [i.e., undertaking feeding migrations from freshwater to the sea and back (Elliott 1994; Jonsson 1985; Jonsson and Jonsson 1993; Nevoux et al. 2019)], with a sex ratio skewed toward females among anadromous and towards males among freshwater resident trout (Bekkevold et al. 2004; Duval et al. 2021; Jonsson 1985; Nevoux et al. 2019). While genetic components in facultative anadromy cannot be ruled out (Ferguson et al. 2016), environmental conditions have been shown to affect the decision to migrate (Olsson et al. 2006; Wysujack et al. 2009), and coexisting anadromous and resident trout interbreed and must be considered components of the same population (Duval et al. 2021; Hindar et al. 1991). This means that even though embryos and juveniles may experience similar temperature regimes in a population, their epigenomes could also be influenced by the different life histories and environmental conditions experienced by their parents.

We analyzed genome‐wide methylation of juvenile brown trout subjected to different incubation temperatures. Based on wild‐caught anadromous and resident trout from the same river system, four types of crosses were established, involving pure anadromous and resident crosses and reciprocal crosses between anadromous and resident males and females. Fertilized eggs were divided into two pools, incubated at ambient temperature or at 3°C above ambient temperature until the time of first feeding, after which all groups were maintained at the same temperature and sampled for analysis ca. 10.5 months after fertilization. Based on whole‐genome bisulphite pooled sequencing, we analyzed methylation in the different experimental groups. We first tested the hypothesis that elevated temperature during incubation would lead to altered methylation that persisted later in life, potentially reflecting developmental phenotypic plasticity. Second, we tested the hypothesis that parental life history (resident vs. anadromous) influences DNA methylation patterns in offspring, potentially affecting temperature‐related differential methylation. Specifically, we expected maternal effects to have a stronger influence than paternal effects, as maternal provisioning via eggs may play a key role in shaping early gene expression and possibly methylation patterns (Harry and Zakas 2023). Finally, we assessed the results in the context of adaptive responses and resilience to climate change.

## 2 Materials and Methods

### 2.1 Crosses and Hatchery‐Rearing

**FIGURE 1:**
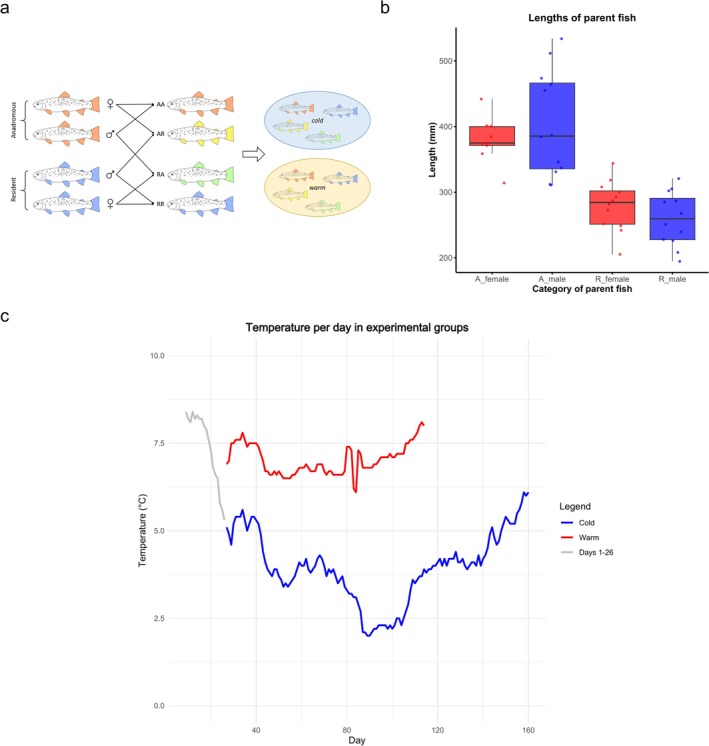
(a) Overview of experimental design, involving crosses between anadromous females and anadromous males (AA), anadromous females and resident males (AR), resident females and anadromous males (RA), and resident females and resident males (RR). Offspring from each type of cross were subsequently divided into two pools maintained in a “cold” environment (ambient water temperature) and “warm” environment (heated by ca. 3°C above ambient temperature). (b) Lengths of parent fish used in the experiment. A denotes anadromous and R denotes resident trout. (c) Temperature per day across the experimental period. From November 1–27 eggs were maintained at the same temperature, whereas from November 27 and until first feeding the “warm” group was maintained at ca. 3^o^C above the natural temperature in the River Imsa.

Fertilization and rearing took place at the Ims Research Station, Norwegian Institute for Nature Research (NINA). Experimental fish were sampled from the River Imsa in southwestern Norway (outlet at 58.903° N, 5.965° E) in autumn 2018. Anadromous spawners were sampled in a fish trap close to the outlet, whereas resident spawners were sampled by electrofishing in the tributary Fossbek Stream ca. 1 km from the outlet. The latter locality has been inaccessible to anadromous trout since 1993 due to the establishment of an experimental barrier, but is assumed to have been a part of the main Imsa population prior to 1993. Crosses were set up on November 1, 2018 using 12 males and 12 females from each of the two groups, although it subsequently became apparent that eggs from three anadromous females were barren. In total, 9 families of anadromous female × anadromous male crosses (hereafter termed AA), 9 families of anadromous female × resident male crosses (AR), 12 families of resident female × anadromous male crosses (RA) and 12 families of resident female × resident male (RR) crosses were established. Approximately 100 eggs were derived from each family and were divided into two groups, one incubated in water piped from the River Imsa, representing ambient temperature (in the following denoted “cold”), whereas the other was incubated in Imsa water heated by ca. 3°C above ambient temperature (denoted “warm”). A total of 8 experimental groups was established (4 types of crosses incubated at two different temperatures). An overview of the experimental design is shown in Figure 1a and sizes of parent fish are shown in Figure 1b.

The heating of water took place from November 27, 2018 to onwards. Prior to this time, ambient water temperature was as high as 8.4°C and further heating would likely have led to high mortality and developmental abnormalities (Realis‐Doyelle et al. 2016). The temperature per day for the two experimental groups is shown in Figure 1c, where the highest temperature during the “warm” treatment was 8.1°C. Until this point of time in the experiment, all families in each temperature treatment were kept in separate compartments, but experienced the same water source, that is, river water that was piped in and either heated or not heated.

At the time of first feeding, offspring were transferred to containers with water at ambient temperature, and individuals from the crosses within each experimental treatment were pooled. This occurred between January 15 and February 22, 2019 for the “warm” group and April 8–10, 2019 for the “cold” group. On September 12, 2019, 20 individuals were sampled from each experimental group, euthanized, and stored individually in 96% ethanol at −18°C until DNA extraction.

### 2.2 DNA Extraction and Whole Genome Bisulphite Sequencing

Fork length was measured for all individuals, and subsequently, ca. 4 × 4 × 4 mm muscle samples were taken from the same place adjacent to the dorsal fin. Extraction of DNA was conducted using the E.Z.N.A DNA Tissue Extraction Kit (Omega Bio‐Tek) according to the manufacturer's recommendations. DNA concentration was subsequently measured for each individual using a NanoDrop instrument (Thermo Fisher Scientific Inc.), and DNA from all individuals from each experimental group was pooled, ensuring equimolar representation from each individual. Whole‐genome‐bisulphite sequencing (WGBS) was outsourced to Novogene Europe (Cambridge). Sequencing was conducted by 150 base pair (bp) paired‐end on the Illumina HiSeq 2500 platform and aimed for a minimum coverage of 80× for each experimental group. We chose pooled WGBS to obtain high coverage and a broad genome‐wide assessment of methylation differences across factorial combinations of parental life history and temperature treatment. While this design precludes formal statistical testing of individual‐level variation, it enables robust detection of consistent group‐level methylation shifts, which was our primary objective.

### 2.3 Read Mapping and Methylation Calling

Reads were mapped to a brown trout reference genome (NCBI accession: GCF_901001165.1), which has a length of 2.372 Gb and has been assembled to the chromosome level, containing 40 chromosomes, 1400 unanchored scaffolds, and one mitogenome (Hansen et al. 2021). The WGBS reads were filtered using Trim Galore v0.6.6 (https://github.com/FelixKrueger/TrimGalore) by allowing “‐‐trim1” and were mapped to the reference genome using Bismark v0.22.3 (Krueger and Andrews 2011). The mapped reads were deduplicated using “deduplicate_bismark.”

We subsequently ran “bismark_methylation_extractor” and “bismark2bedGraph” to extract all the sequenced CpG sites together with their methylation status. The information was stored in the COVERAGE files in the output. During the extraction process, the first two base pairs of all the Read 2 files were removed based on the M‐bias plots. As CpG is palindromic and complementary CpGs are synchronized in methylation due to dnmt1 activity during DNA replication, complementary CpGs were merged. The COVERAGE files of all eight samples were merged using a custom script. This generated a file where the CpGs of all the samples were aligned by coordinate. Within each sample, the CpGs with coverage lower than 15 were marked as missing. CpGs with overall coverage larger than 800 were removed. The coverage thresholds were decided from the coverage distribution (Figure S1). CpGs containing missing values were filtered out.

To obtain an overview of methylation variation across the samples, we conducted a principal component analysis (PCA) and a clustering analysis. The PCA was implemented using the “prcomp” function in R, and the clustering analysis using the “pheatmap” package in R.

### 2.4 Methylation Response to the Temperature Treatments

We adopted two different methods to test the methylation response of brown trout to the temperature treatments. In our first approach, we tested the methylation response within offspring of each cross type and made comparisons among them. For each cross type, we calculated the methylation difference per CpG between the two temperature treatments. A threshold of 0.35 was used to define CpG outliers. The threshold was determined to keep the number of outliers lower than 0.1% of the total number of CpGs recovered. A threshold lower than this would result in an excessive number of outliers and increase the noise in detecting differentially methylated regions (DMRs; see below). Therefore, four sets of outliers were obtained corresponding to the cross types. We ran PCA on each set to see if the outliers would segregate the samples from the other cross types according to the temperature treatments, which would indicate that different cross types share consistent mechanisms of methylation when responding to the temperature treatments.

We subsequently split each outlier set to hyper‐ and hypomethylated subsets with respect to warm temperature and treated each subset separately for the following comparisons. First, we inspected the overlap of the outliers among the four cross types through a Venn diagram using the “ggvenn” package in R. Second, we examined the distribution of the outliers across the genome and identified DMRs. Genes surrounding the DMRs within a distance of 10 kb were extracted and compared among the offspring types using a Venn diagram. The 10‐kb threshold was decided according to the median length of the intergenic regions (Figure S2). Third, we ran GO term enrichment analysis for each gene set and inspected the overlaps among the significant GO terms from different offspring types.

In the second method, we assessed the overall effects of the temperature treatments regardless of the offspring types. This was achieved by applying PST combined with the overall methylation difference as described in Liu et al. (2022). PST is a measure of phenotypic differentiation between groups, comparable to *F*_ST_ (Leinonen et al. 2013; Pujol et al. 2008). Here, we regarded the methylation level of each CpG as a phenotype and divided the eight experimental groups into two groups corresponding to the cold and warm treatments. PST was calculated using a custom script in R, and the methylation difference was represented by the difference between the two groups in average methylation level. CpGs with PST > 0.8 and with a methylation difference > 0.2 were considered to be outliers. The outliers were split into hyper‐ and hypomethylated subsets with respect to warm temperature, and the two subsets were treated separately. We inspected the distribution of the outliers across the genome and identified DMRs. For each DMR, we zoomed into the genomic region situated within 10 kb up‐ and downstream of it and visually verified the DMR. We also examined the genes residing in the zoom‐in region.

### 2.5 Influence of Parental Life History on the Methylation Pattern

We examined the parental influence on methylation using the same PST‐based method as implemented above, dividing the samples into anadromous (AA and AR from both cold and warm treatments) and resident (RR and RA from both cold and warm treatments) groups according to the maternal life history (see Figure 1a). For comparison, we also conducted the same analysis according to the paternal life history, hence dividing the samples into anadromous (AA and RA from both cold and warm treatments) and resident (RR and AR from both cold and warm treatments) groups according to the paternal life history.

### 2.6 Defining DMRs From Outliers

The outliers in this study represent differentially methylated CpGs. When a group of outliers is densely clustered in the genome, this region is defined as a differentially methylated region (DMR). We identified DMRs by first plotting the density of the outliers across the genome using non‐overlapping 1‐kb windows. Any window that contained a minimum of five outliers was defined as a DMR.

The 1‐kb window size and the density threshold (five outliers minimum) were determined by studying the properties of the lowly methylated regions (LMRs) in the brown trout genome. Like all other vertebrates investigated so far, the brown trout genome is globally methylated. CpGs with low methylation levels, therefore, play vital roles in activating gene expression (Brenet et al. 2011; Jones 2012; Liu et al. 2022). We inspected the genome‐wide distribution pattern of CpGs with various methylation levels and found that CpGs with low methylation levels tended to form dense clusters (Figure S3). We defined these clusters as LMRs and identified them by grouping neighboring lowly‐methylated CpGs (methylation level < 0.05) that were closer than 200 bp (it is the boundary between the two observed peaks in the first plot of Figure S3). We then examined the length distribution of the LMRs and found that the length peaked at ca. 1 kb (Figure S4). Therefore, 1 kb was used as the window size for scrutinizing the outlier density across the genome. Because 200 bp was used as the maximum distance between neighboring lowly‐methylated CpGs in an LMR, a 1‐kb LMR would contain at least five lowly‐methylated CpGs. Therefore, five was used as the outlier density threshold for defining DMRs.

We took two additional measures to make sure we obtained high‐quality DMRs. First, as an excessive number of outliers will generate high‐density regions by random chance, thereby increasing noise in DMR detection, we intentionally limited the number of outliers from each analysis to below 0.1% of the total number of CpGs recovered. Second, each DMR was visually confirmed by generating a zoom‐in view of the region surrounding the DMR (a range of 10 kb up‐ and downstream).

### 2.7 GO Term Enrichment Analysis

The gene lists obtained from the analyses were tested for GO (gene ontology) term enrichment using the “weight01” algorithm of the “topGO” package in R (Alexa and Rahnenfuhrer 2021). The GO IDs of the genes were retrieved by blasting the genes against the Swiss‐Prot database (Release 2021_03, The UniProt Consortium 2019).

## 3 Results

### 3.1 Lengths and Weights of Offspring

**FIGURE 2:**
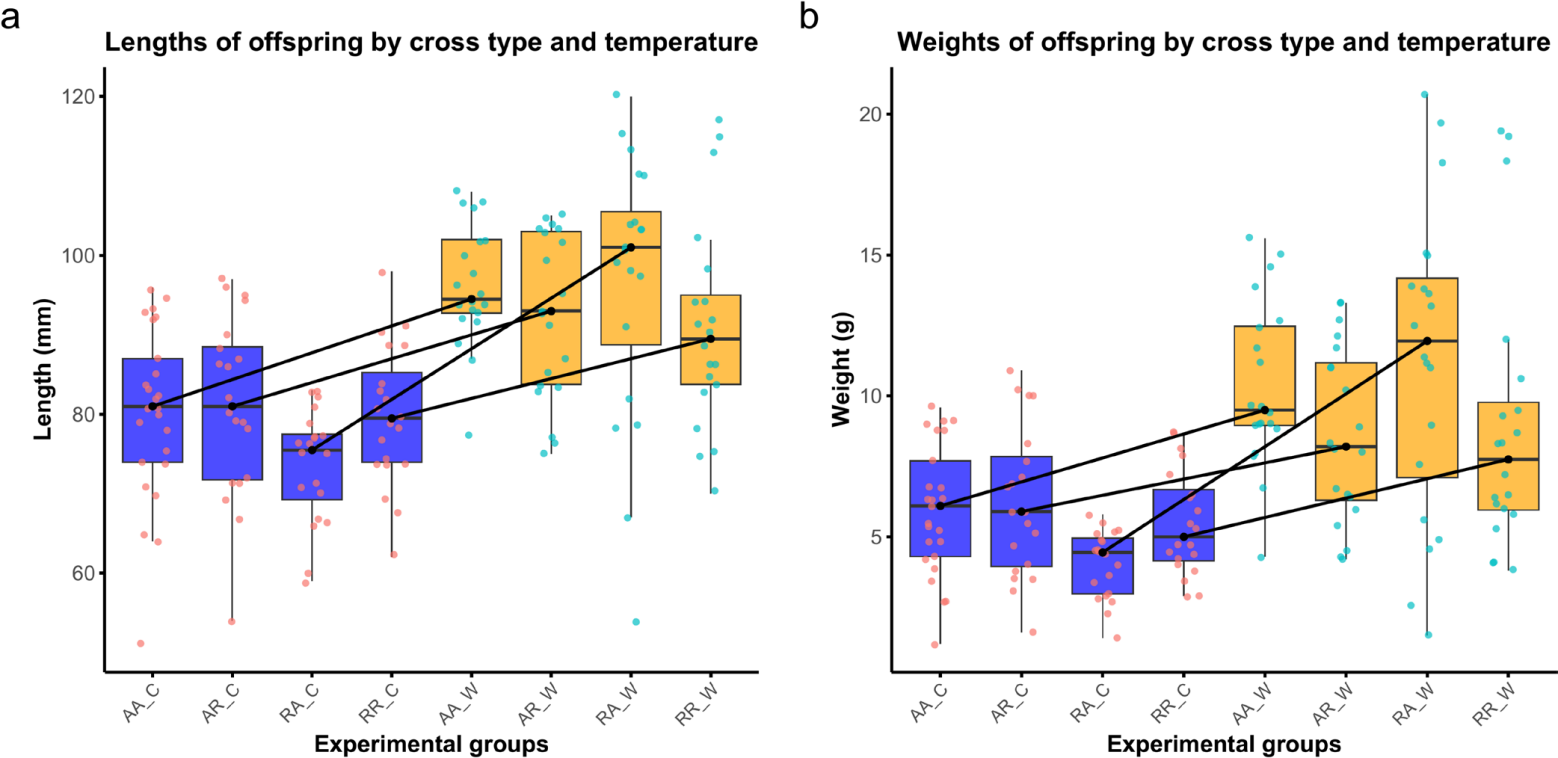
(a) Boxplot showing lengths of offspring from different experimental crosses and treatments. (b) Boxplot showing weights of offspring from different experimental crosses and treatments. C denotes cold and W warm treatments. The directions of change between different treatments for each cross type are indicated by lines.

The lengths of offspring from the different cross types reared under different temperature regimes are shown in Figure 2a and weights in Figure 2b. Body length was significantly affected by temperature (ANOVA *F*_1,157_ = 77.75, *p* < 0.001). In contrast, cross type had no significant effect on length (ANOVA *F*_3,157_ = 0.43, *p* = 0.729), whereas the interaction between cross type and temperature was marginally non‐significant (ANOVA *F*_3,157_ = 2.49, *p* = 0.062), suggesting a potential but weak variation in temperature effects among different genetic crosses. Log‐transformed weight (to improve normality and homogeneity of variance) was significantly affected by temperature (ANOVA *F*_1,157_ = 65.06, *p* < 0.001), but not by cross type (ANOVA *F*_3,157_ = 0.996, *p* = 0.396). The interaction between cross type and temperature was significant (ANOVA *F*_3,157_ = 2.87, *p* = 0.038), suggesting that offspring from different crosses responded differently to temperature for this trait. The interaction between cross type and temperature was primarily driven by offspring of resident mothers and anadromous fathers, which showed a greater increase in length and weight at warm temperatures compared to other cross types (Figure 2a,b).

### 3.2 Overview of Methylation Variation

A total of 33,821,316 CpG sites were recovered, accounting for 79.7% of all CpG sites in the reference genome. The sequencing coverage for each pooled sample ranged between 46 and 57, with an average of 52 (Table S1). The overall methylation level of the genome was highly consistent across pooled samples, ranging from 0.764 to 0.777, with a mean of 0.768 (Table S1).

**FIGURE 3:**
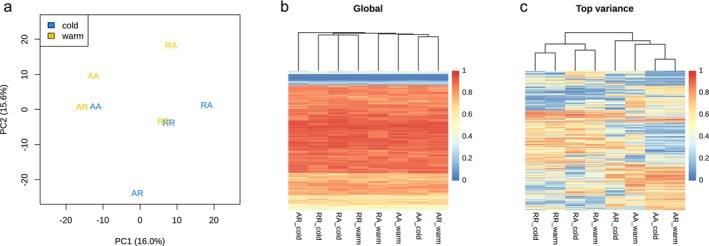
Methylation variation across the samples. (a) PCA of the eight samples based on all sequenced CpGs. The numbers in the brackets are the percentages of the total variance explained by the corresponding PCs. (b) Clustered heatmap using 2000 random CpGs. (c) Clustered heatmap using the top 2000 CpGs in methylation variance.

The genome‐wide PCA of methylation revealed little tendency for forming subgroups among the samples, although the first axis separated the samples according to the maternal lines, implicating an influence from the maternal life history (Figure 3a). The eigenvalues were rather evenly distributed across the axes, suggesting that the overall variance contained limited amounts of meaningful information, which was also evident when considering other axes. A clustering analysis based on a global subset of 2000 randomly chosen CpGs concurred with the PCA result (Figure 3b). Hence, the internal branches were extremely short, indicating no sub‐structure. In contrast, a clustering analysis based on the top 2000 CpGs in methylation variance split the samples into two groups consistent with maternal life history (Figure 3c). This suggests that the most variable CpGs among the samples are under the influence of the maternal life history.

### 3.3 Methylation Response to the Temperature Treatments

**FIGURE 4:**
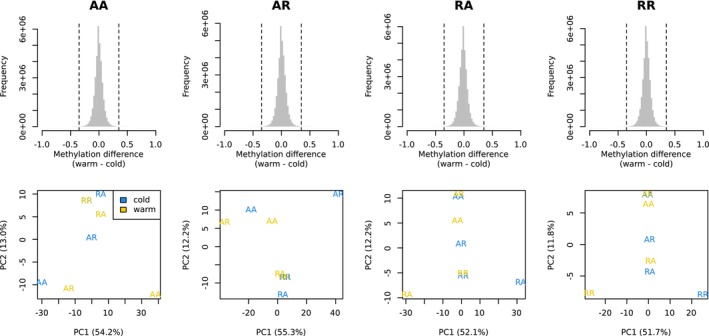
Outliers within each offspring type based on the methylation difference between the two temperature treatments. Upper panel: Distribution of methylation difference between temperature treatments in each offspring type. The dashed vertical lines are the thresholds for outliers. Lower panel: PCA based on the outliers from each offspring type.

**FIGURE 5:**
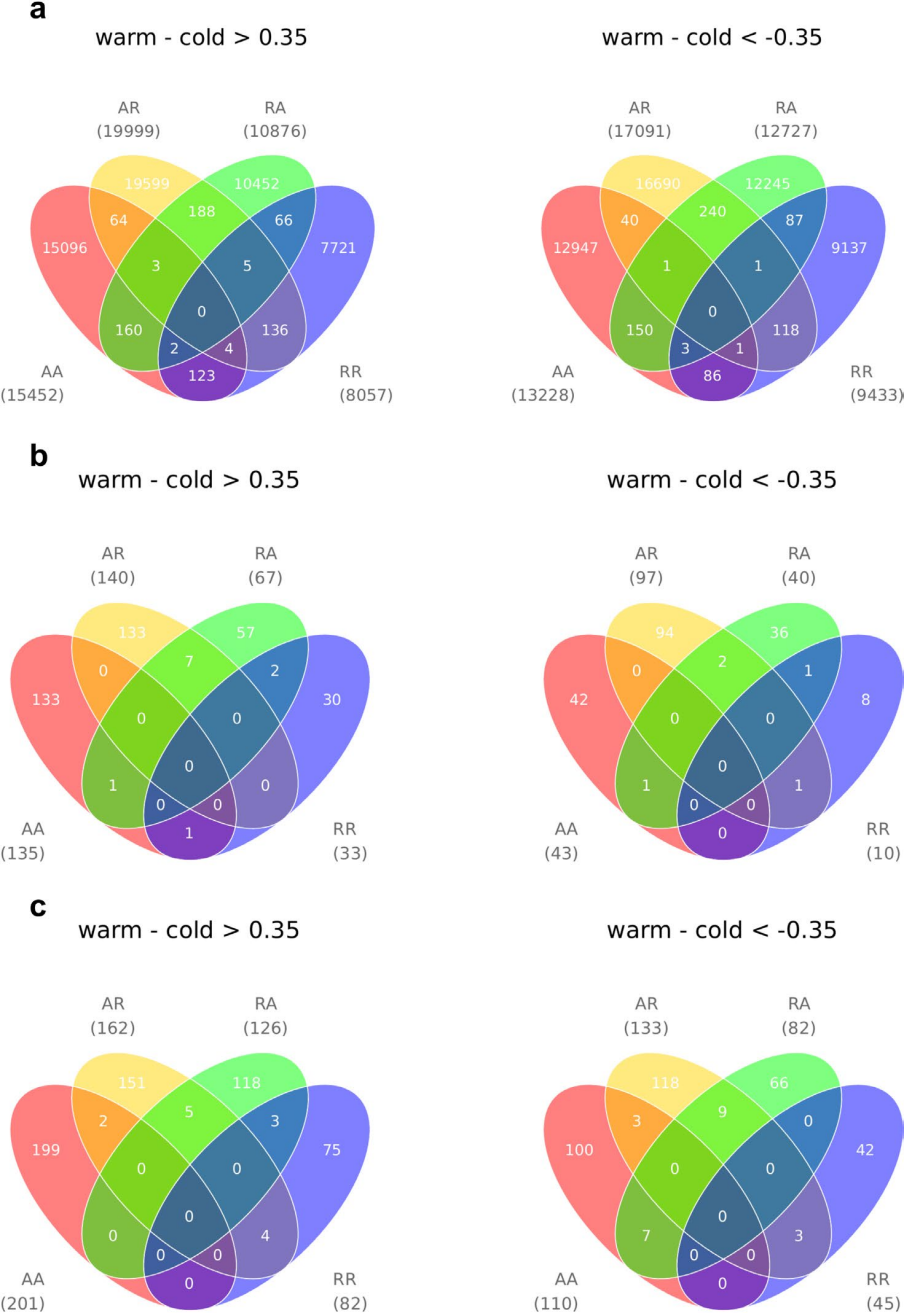
(a) Venn diagrams of the outliers from Figure 4. The outliers in each offspring type are those differentially methylated between temperature treatments. The outliers in each offspring type were separated into two subsets corresponding to those hypermethylated in warm (left panel) and cold (right panel) treatments, respectively. Numbers in the brackets are the numbers of outliers. (b) Venn diagrams of the genes surrounding the DMRs detected by Figure 6 (listed in Table S2). The numbers in the brackets are the number of genes. (c) Venn diagrams of the significant (*p*‐value < 0.05) GO terms upon running GO term enrichment tests on the genes from (b). The numbers in brackets are the number of significant GO terms.

**FIGURE 6:**
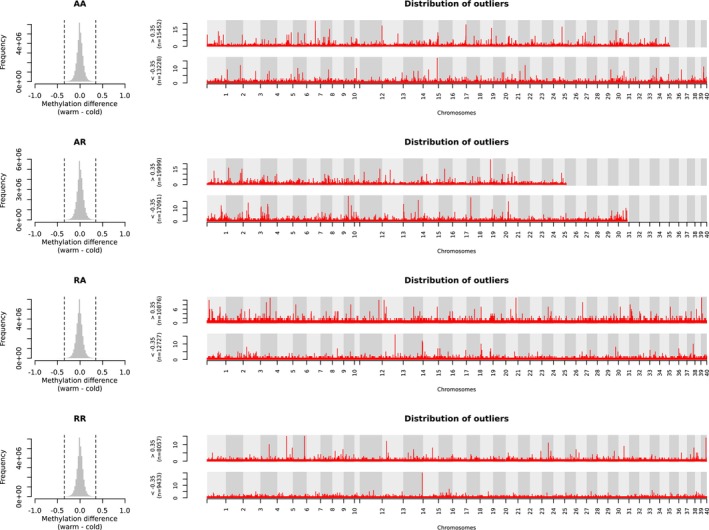
Distribution of outliers across the genome. The outliers are from Figure 4 and are those differentially methylated between temperature treatments in each offspring type. The *y*‐axes of the Manhattan plots represent the number of outliers in non‐overlapping 1‐kb sliding windows across the genome. *y* > = 5 defines DMRs (listed in Table S2).

In the first of our two different methods to test the methylation response of brown trout to the temperature treatments, we tested the methylation response within each cross type and made comparisons among them. Using a threshold of 0.35 for methylation difference, we identified 28,680, 37,090, 23,603, and 17,490 CpG outliers for AA, AR, RA, and RR, respectively. PCA for each set of outliers found none of the sets to separate samples according to the temperature treatments (Figure 4), suggesting that the CpG outliers did not show a universal temperature response across types of crosses. When comparing the four sets of outliers (Figure 5a), we still did not find any outliers being shared by all offspring types. By examining the distribution of the outliers across the genome, we identified differentially methylated regions (DMRs) (Figure 6) and extracted the genes surrounding the DMRs (Table S2). This involved 481 DMRs, the surrounding regions of which contained 525 protein‐coding genes and also 54 lncRNAs (long non‐coding RNAs). No genes were found to be shared by all cross types (Figure 5b). We conducted GO term enrichment analysis for each gene set (Table S3) and inspected the overlaps among the significant GO terms from different offspring types (Figure 5c). No significant GO terms were shared by all cross types.

Although the comparisons among the cross types did not find a common methylation response to the temperature treatments, some other patterns were observed. For example, the warm treatment consistently resulted in more hypermethylated genes than the cold treatment (Figure 5b), likely causing more genes to be repressed. In addition, RR appeared least responsive to the temperature treatments as it exhibited the lowest number of differentially methylated genes (Figure 5b).

**FIGURE 7:**
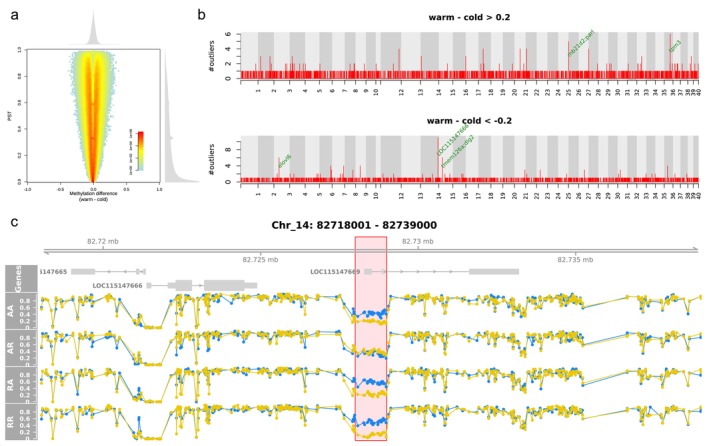
Detection of methylation response to the temperature treatments using PST and overall methylation difference. (a) Joint distribution of PST and overall methylation difference between the temperature treatments. (b) Distribution of outliers across the genome. The *y*‐axes of the Manhattan plots represent the number of outliers in non‐overlapping 1‐kb sliding windows across the genome. Green labels next to the peaks specify the genes surrounding the DMRs. (c) A zoom‐in view of the DMR showing the strongest signal in (b). The blue dots indicate the methylation levels of CpGs in the cold treatment, and the yellow ones for the warm treatment. The red box highlights the DMR.

**FIGURE 8:**
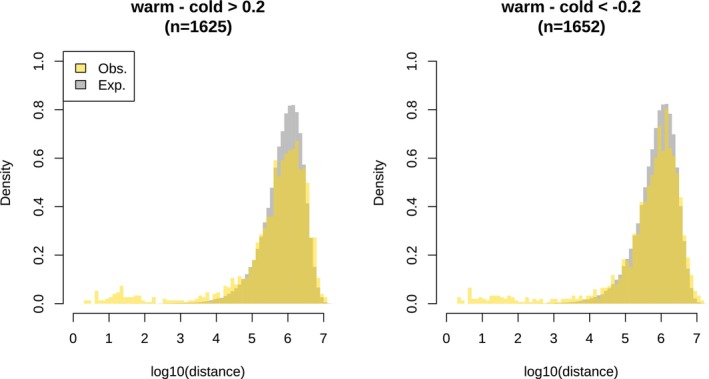
Distribution of the neighboring distance among the outliers obtained from Figure 7. The numbers in brackets are the number of outliers.

**FIGURE 9:**
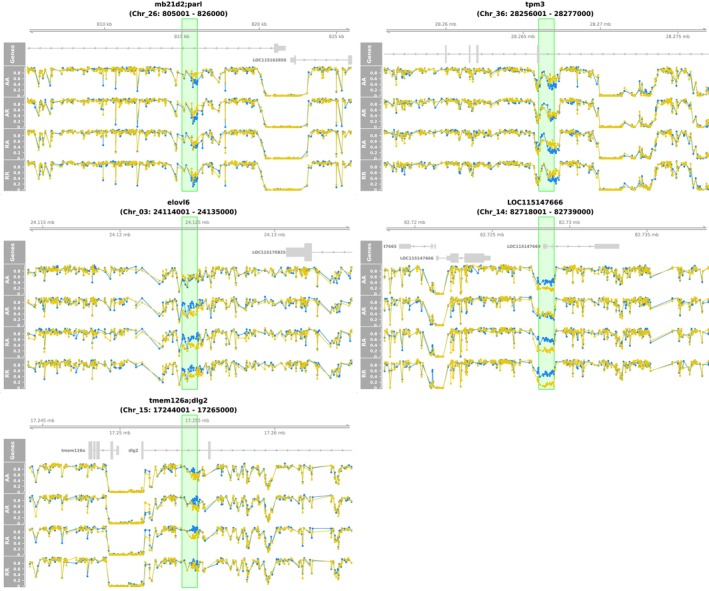
Zoom‐in views of the DMRs in Figure 7b. The blue dots indicate the methylation levels of CpGs in the cold treatment, and the yellow dots in the warm treatment. The green boxes represent the DMRs. Each plot provides a view of 10 kb upstream and downstream of the DMR. The header specifies the protein‐coding genes neighboring the DMR and the coordinate of the viewed region.

The second method was based on PST and overall methylation difference (Figure 7a). It considered the overall difference between the temperature treatments regardless of the cross type. Using a threshold of 0.2 for the methylation difference and 0.8 for PST, we identified 3277 outliers, of which 1625 and 1652 were hyper‐ and hypomethylated, respectively, in the warm treatment. The outliers tended to be randomly distributed in the genome (Figure 8), indicating low functional significance. Indeed, upon examining the distribution of the outliers across the genome, we only identified five DMRs (Figure 7b). A DMR near the end of Chr_14 exhibited the strongest signal, and a zoom‐in view confirmed its validity (Figure 7c). This DMR was hypomethylated in the warm treatment for AA, RA and RR. However, in AR, the methylation level did not differ between the temperature treatments. This DMR was in close proximity to a protein‐coding gene named LOC115147666, ca. 2.5 kb downstream of the gene. This gene is annotated as “gastrula zinc finger protein XlCGF26.1‐like,” an uncharacterized gene. However, this DMR was in even closer proximity to a lncRNA (long non‐coding RNA) named LOC115147669, right at the beginning of this lncRNA. We also looked into the other four DMRs (Figure 9). Six genes were found in the proximal regions, that is, mb21d2, parl, tpm3, elovl6, tmem126a, and dlg2. However, the methylation difference between the temperature treatments was relatively weak.

### 3.4 Influence of Parental Life History on the Methylation Pattern

**FIGURE 10:**
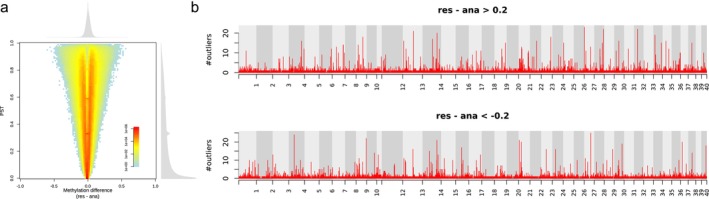
Influence of the maternal life history on the methylation pattern of the offspring. (a) Joint distribution of PST and overall methylation difference between the groups with different maternal life histories. (b) Distribution of outliers across the genome. The *y*‐axes of the Manhattan plots represent the number of outliers in non‐overlapping 10‐kb sliding windows across the genome.

**FIGURE 11:**
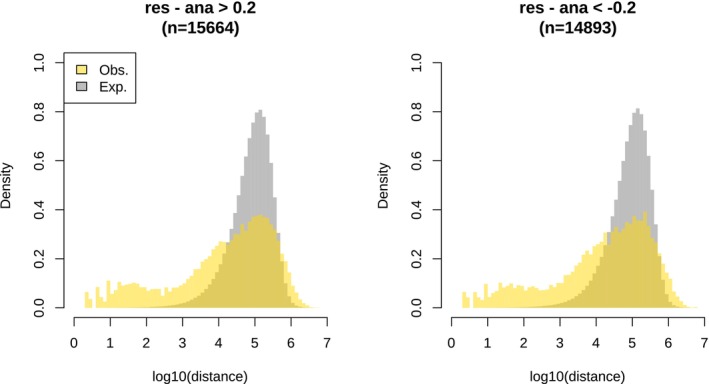
Distribution of the neighboring distance among the outliers obtained from Figure 10 (resident vs. anadromous mothers). The numbers in brackets denote the number of outliers.

Since the genome‐wide PCA and clustering analysis indicated an influence of maternal life history on the general methylation pattern of the offspring, we examined this influence using the same PST‐based method used above, dividing the samples into maternal anadromous (AA and AR from both cold and warm treatments) and maternal resident (RR and RA from both cold and warm treatments) groups. The distribution of the methylation difference due to maternal life history (Figure 10a) exhibited larger dispersion than the distribution due to the temperature treatments (Figure 7a). Using the same thresholds for the methylation difference and PST, we identified 30,557 outliers, of which 15,664 and 14,893 were hyper‐ and hypomethylated, respectively, in the resident maternal group (Figure 11). The outliers exhibited a high tendency to form clusters, indicating high functional significance. Upon examining the distribution of the outliers across the genome, we identified 267 DMRs (139 and 128 being hyper‐ and hypomethylated, respectively, in the resident maternal group) (Figure 10b; Table S4). A total of 335 protein‐coding genes was found surrounding these DMRs. GO term enrichment analyses indicated enrichment of genes particularly related to nucleotide metabolism, the nervous system, blood coagulation, and digestion (Table S5).

**FIGURE 12:**
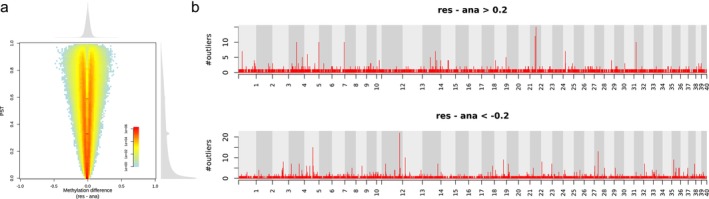
Influence of the paternal life history on the methylation pattern of the offspring. (a) Joint distribution of PST and overall methylation difference between the groups with different paternal life histories. (b) Distribution of outliers across the genome. The *y*‐axes of the Manhattan plots represent the number of outliers in non‐overlapping 10‐kb sliding windows across the genome.

**FIGURE 13:**
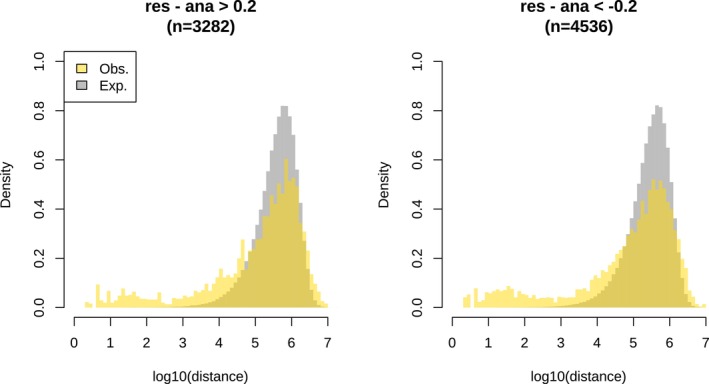
Distribution of the neighboring distance among outliers obtained from Figure 12 (resident vs. anadromous fathers). The numbers in brackets denote the number of outliers.

We conducted the same type of analysis that we did for maternal life history for paternal life history. Hence, we divided the samples into anadromous (AA and RA from both cold and warm treatments) and resident (RR and AR from both cold and warm treatments) groups according to paternal life history (Figure 12a). We identified 7818 outliers, of which 3282 and 4536 were hyper‐ and hypomethylated, respectively, in the group with resident fathers (Figure 13). The outliers exhibited a moderate level of clustering tendency (Figure 13) and resulted in 46 DMRs (13 and 33 being hyper‐ and hypomethylated, respectively, in the group with resident fathers) (Figure 12b; Table S6). A total of 55 genes were found surrounding these DMRs. GO term enrichment analyses showed an enrichment of genes particularly associated with oxidative stress, the nervous system, and immunity (Table S7).

## 4 Discussion

Our study provided new insights on temperature‐induced methylation responses in a fish species expected to be impacted by climate change (Borgwardt et al. 2020; Jonsson and Jonsson 2009). As a whole, the temperature treatment in the early life stage showed only weak effects on the methylation pattern in later life, and there was very little consistency in temperature‐related CpG outliers across different crosses. Instead, the results showed that the largest contributor to differential methylation was parental, with maternal exceeding paternal contributions. In the following, we discuss our experimental approach, the methylation response to different temperature regimes, the influence of parental life history on methylation patterns, and the significance of the results in a climate change perspective.

### 4.1 Experimental Approach

Some aspects of the experimental setup need specific consideration. First, due to high initial water temperature, the first 27 days of incubation involved the same temperatures for the two experimental groups, precluding temperature‐related differential methylation responses in the initial stages of development. The experiment therefore could not capture possible differential methylation occurring in the very earliest phases of development. A previous study of European grayling ( *Thymallus thymallus* ) did uncover significant methylation differences between embryos incubated at different temperatures following fertilization (Savilammi et al. 2021). However, methylation differences were not tested at later stages, so it remains unknown if the differences were persistent. In a study of brook charr by Venney et al. (2022), eggs were incubated at different temperatures directly after fertilization, and the offspring were maintained at different temperatures until first feeding, after which the fish were transferred to a common temperature regime and subsequently analyzed 2 months later. In this case, negligible differences in methylation due to the temperature treatments were observed, whereas the temperatures experienced by parents had a major effect on offspring methylation. The design of this study, involving analysis of methylation a considerable time after the temperature treatment, is comparable to our study. As the results of Venney et al. (2022) are qualitatively similar to ours, it is plausible that different incubation temperatures immediately after incubation in our study would not have led to different methylation later in life.

Second, we made the choice to sample and analyze individuals of the same chronological age, even though there were considerable differences in lengths of individuals from the two temperature treatments. Chronological age has a major effect on methylation (Horvath and Raj 2018), also in fishes (Anastasiadi and Piferrer 2020; Liu et al. 2022; Mayne et al. 2021), and if we had sampled fish of similar length, they would be of different age, possibly inducing methylation differences. On the other hand, sampling individuals of the same age but different lengths involves the risk of detecting methylation differences that reflect differences in developmental stage rather than direct temperature effects. As it turned out, there were very small differences in methylation between temperature treatments, which suggests this to be a minor issue, although it cannot be ruled out that the few differences observed between treatments could reflect developmental stage rather than temperature.

Third, methylation is tissue‐specific. We made the choice to focus on muscle tissue due to its biological relevance at the juvenile stage: first feeding is a period of high mortality, and individual survival depends critically on muscle‐driven activity related to foraging, territory establishment, and predator avoidance (Elliott 1994). Muscle tissue has been analyzed in other studies of methylation in different fish species (Le Luyer et al. 2017; Metzger and Schulte 2017), but there are also examples of using, for example, liver (Venney et al. 2024, 2022) or whole embryos (Savilammi et al. 2021; Venney et al. 2020). The use of different tissues should be considered as a complicating factor when comparing results across different studies, and ideally analysis of multiple tissues would have strengthened the conclusions of our study.

Finally, to mimic the natural situation, we used parent fish sampled from the wild rather than experimental fish reared in a common environment and subjected to specific treatments. This means that the specific environmental conditions experienced by the individuals differed along a number of dimensions, including temperature, salinity exposure, and diet composition. Hence, it would be difficult to ascribe parental components of differential methylation to specific environmental conditions, such as temperature.

### 4.2 Methylation Response to the Temperature Treatments

No strong, consistent temperature‐induced methylation was found across offspring of different crosses. In fact, one could question if there was any methylation response to temperature, or alternatively if methylation had faded over time, which would indicate that methylation changes were either reversible or not stably maintained across developmental stages. We argue, however, that temperature incurred more subtle long‐lasting effects on methylation, as the warm treatment consistently yielded more hypermethylated genes than the cold treatment. This is also the direction of change observed in studies of both threespine stickleback ( *Gasterosteus aculeatus* ) and European grayling (Metzger and Schulte 2017; Savilammi et al. 2021), although other studies of brook charr, turbot (*Scopthalmus maximus*), and Atlantic cod ( *Gadus morhua* ) reported either negligible change of methylation or gene‐specific patterns of hypo‐ and hypermethylation (Puvanendran et al. 2023; Suarez‐Bregua et al. 2020; Venney et al. 2022), and a study of Atlantic herring showed the opposite pattern of hypomethylation with increasing temperature (Kho et al. 2024). Hypermethylation at higher temperatures in the present study could reflect prevention of excessive transcriptional activity in response to thermal stress, or transcriptional regulation by silencing non‐essential genes or modulating expression to regulate physiological responses to high temperature (Metzger and Schulte 2017), but the overall weak patterns preclude a more definitive interpretation.

It is also possible that offspring of different crosses use different methylation mechanisms in response to temperature. If so, we might expect to see common physiological responses, albeit mediated by different methylation. However, the lack of overlap of GO terms for differentially methylated genes in different crosses did not point in this direction. On the other hand, the finding that offspring of RR crosses showed very low numbers of differentially methylated genes, whereas the other cross types (especially AR) exhibited much higher numbers could suggest a difference in responses that also involves different mechanisms.

Using our PST‐based approach for detecting overall methylation differences between temperature treatments regardless of offspring types, it showed the most pronounced DMR signal to be closest to an lncRNA (long non‐coding RNA) locus. Furthermore, a total of 54 lncRNAs was represented among DMRs associated with specific offspring types, although none were shared by all types. lncRNAs have multiple functions in regulating gene expression by fine‐tuning chromatin structure, interacting with transcription factors, and affecting the stability and translation of RNA (Mattick et al. 2023). Specifically, with respect to temperature, examples exist of lncRNAs regulating heat shock proteins (Ji et al. 2021), making it plausible that the DMRs associated with lncRNAs in the present study represented temperature‐related responses.

### 4.3 Parental Influence on Methylation Patterns

Our study found that parental life history had a significant influence on offspring DNA methylation patterns, exceeding the effects of early‐life incubation temperature. This is consistent with previous findings in brook charr and Atlantic cod (Venney et al. 2022), where elevated temperature experienced by juveniles had negligible effects on methylation later in life, whereas temperature experienced by parents during sexual maturation affected methylation in offspring, potentially leading to cross‐generational phenotypic plasticity. The latter two studies used parental fish reared in a controlled environment where temperature was manipulated, whereas in our study, parent fish were derived from uncontrolled wild environments. Also, they analyzed methylation in both parents and offspring, whereas our study only examined offspring. Hence, compared to Venney et al. (2022) and Puvanendran et al. (2023) our study does not strictly demonstrate inheritance of methylation but only significant parental contributions to methylation, and the parental contributions to methylation likely reflect a wide range of environmental conditions, with temperature just being one of them.

The results showed a particularly strong maternal contribution, while paternal effects, although present, were weaker. The maternal contribution could represent either direct transfer of methylation or more indirect maternal effects, that is, influence methylation through maternally derived molecules in the egg, whereas the paternal effects must be assumed to represent more direct inheritance of methylation (Venney et al. 2020; Wellband et al. 2021). While there was some overlap of enriched GO terms for genes showing differential methylation due to maternal and paternal contribution, respectively, GO terms related to nucleotide metabolism and digestion were primarily enriched in maternally contributed methylation differences. This would be consistent with transfer of nutrients and molecules in eggs that affect methylation.

The question arises if the parentally transmitted methylation differences reflect the different environmental conditions experienced by resident and anadromous trout from the study population or more intrinsic differences between the two life history types. Baerwald et al. (2016) have previously provided evidence for methylation differences between migratory and resident rainbow trout ( *Oncorhynchus mykiss* ), but it was not clear if the differences were a cause or an effect of life history differences. In the present study, the many functionally different genes showing differential methylation suggest that broad environmental differences alone could underlie the results. However, if a few key regulators of life history strategy are differentially methylated, they could act as trans‐acting factors, triggering cascades of gene expression changes that ultimately differentiate resident and anadromous phenotypes. An extended experimental setup involving resident and anadromous trout from several different populations could shed further light on this issue and contribute to resolving the long‐standing debate of ecological, genetic, and epigenetic factors affecting life‐history variation in brown trout and other salmonids (Nevoux et al. 2019).

### 4.4 Implications for Climate Change Responses

The weak and inconsistent temperature‐induced methylation responses observed in our study suggest that early‐life thermal conditions exert limited long‐term effects on methylation. This raises questions about the role of developmental plasticity—at least to the extent it is modulated by methylation—in facilitating responses to a warming climate in the study species. It should be noted that this conclusion only holds for the temperature ranges encompassed by the experiment, where the highest temperature during the “warm” treatment was 8.1°C. This temperature cannot be considered stressful for brown trout, although Jensen et al. (2008) and Meier et al. (2014) showed that an incubation temperature of 8°C had important effects on early life‐history traits and gene expression as compared to a temperature of 5°C.

The experimental setup encompassed intake of water from the home river of the parental fish, hence reflecting the temperature regimes that the fish would experience under natural circumstances. Elevation of the temperature by ca. 3°C also represents a realistic future temperature regime as a result of climate change. Hence, different emission scenarios lead to the prediction of an increase in annual mean temperature ranging from 1.2°C to 3.9°C for this region of Norway by 2100, with increased winter temperatures being most pronounced (Hanssen‐Bauer et al. 2017). However, more extreme fluctuations of temperature are also predicted, including periods of substantially warmer temperatures during winter. If that would lead to higher amplitudes of temperature than experienced in our simulated climate change regime, then we cannot rule out that such higher and potentially stressful temperatures could have caused long‐term effects on methylation.

In contrast to low intragenerational methylation effects, the strong influence of parental life history on offspring methylation suggests that transgenerational epigenetic mechanisms could play a greater role in mediating responses to environmental change, as also suggested in other recent studies of different fish species (Puvanendran et al. 2023; Venney et al. 2022). If parentally transmitted methylation reflects environmental cues experienced during maturation, then this could represent a sort of predictive epigenetic memory that prepares offspring for expected environmental conditions. This could be adaptive, and overall environmental cues perceived by parents could be seen as more predictive than cues perceived by offspring in early life. However, it assumes environmental stability and predictability across generations (Sheriff and Love 2013), a condition that might be met at present but could change in a future environment characterized by a more fluctuating and unpredictable climate. A misalignment between temperature‐related methylation imprints and the temperature conditions that are actually experienced could have serious consequences, particularly for species showing facultative anadromy like the brown trout, where temperature is a key driver of growth and migratory behavior (Jonsson and Jonsson 2016; Nevoux et al. 2019).

Overall, there is considerable evidence for temperature affecting developmental plasticity in a range of organisms, as well as several examples of environmentally induced change of methylation affecting this (Jonsson et al. 2022). However, the present study and related papers (Puvanendran et al. 2023; Venney et al. 2022) show that the outcomes and underlying mechanisms are complex and that in some systems transgenerational effects on methylation may be more important than intragenerational effects. This ultimately reflects the overall complexity of epigenetic and methylation mechanisms, including the degree to which methylation can be inherited across generations. This differs among vertebrates and even among different fish species (Anastasiadi, Venney, et al. 2021; Ortega‐Recalde et al. 2019; Skvortsova et al. 2019; Venney et al. 2020; Wang and Bhandari 2020; Wellband et al. 2021). It would further increase our understanding of the role of methylation in climate change responses if we could obtain more knowledge about transgenerational inheritance of methylation across taxa and whether phylogenetic patterns or constraints influence this process. Finally, study designs like ours, which are based on natural temperature profiles, provide a realistic forecast of future climatic conditions, at least in the short term. Nevertheless, it would be valuable to complement such studies with investigations into methylation changes under more extreme temperature conditions, both to capture the full spectrum of possible epigenetic responses and to better anticipate the effects of an increasing frequency of extreme temperature events due to accelerating climate change.

## Author Contributions

**Shenglin Liu:** conceptualization (equal), formal analysis (lead), investigation (equal), methodology (equal), visualization (equal), writing – original draft (lead), writing – review and editing (equal). **Bror Jonsson:** conceptualization (equal), methodology (equal), project administration (equal), resources (equal), writing – review and editing (supporting). **Larry Greenberg:** conceptualization (equal), investigation (equal), methodology (equal), project administration (equal), writing – review and editing (supporting). **Michael M. Hansen:** conceptualization (equal), formal analysis (supporting), funding acquisition (equal), investigation (equal), methodology (equal), project administration (equal), resources (equal), supervision (equal), visualization (supporting), writing – original draft (equal), writing – review and editing (equal).

## Conflicts of Interest

The authors declare no conflicts of interest.

## Supporting information


**Figures S1‐S4:** ece372154‐sup‐0001‐FigureS1‐S4.pdf.

**Tables S1–S7:** ece372154‐sup‐0002‐TableS1‐S7.xlsx.

## Data Availability

Raw data files for whole‐genome bisulphite sequencing (WGBS) are available at NCBI (The National Center for Biotechnology Information) with BioProject ID PRJNA1229465. Data files with methylated sites are available in DRYAD, https://doi.org/10.5061/dryad.612jm64hn (Liu et al. 2025). Custom scripts for merging COVERAGE files for multiple samples and for calculating PST are available from https://github.com/shenglin‐liu/align_bismark_coverage and https://github.com/shenglin‐liu/PST_for_methylation, respectively.
