# Supplementary material for: Stronger Parental Than Temperature Effects on Methylation in Juvenile Brown Trout (Salmo trutta)
Source: Ecol Evol. 2025 Sep 10;15(9):e72154. doi: 10.1002/ece3.72154 (PMC12422787; doi:10.1002/ece3.72154)

Supplementary figures for

Stronger parental than temperature effects on methylation in juvenile brown trout (*Salmo trutta*)

Shenglin Liu[1§], Bror Jonsson[2], Larry Greenberg[3] and Michael M. Hansen[1§]

[1] Department of Biology, Aarhus University, Aarhus C, Denmark

[2] Norwegian Institute for Nature Research, Sognsveien 68, 0855 Oslo, Norway

[3] River Ecology and Management Group, Department of Environmental and Life Sciences, Karlstad University, Karlstad, Sweden

[§] Corresponding authors:

Shenglin Liu, Department of Molecular Medicine, Aarhus University Hospital, Palle Juul-Jensens Boulevard 99. DK-8200 Aarhus N, Denmark, liushenglin1222@gmail.com

Michael M. Hansen, Department of Biology, Aarhus University, Ny Munkegade 114-116, DK-8000 Aarhus C, Denmark, mmh@bio.au.dk

Fig. S1. Coverage distribution of the sequenced CpGs. Ana, Res, AxR and RxA correspond to AA, RR, AR and RA in the main text.

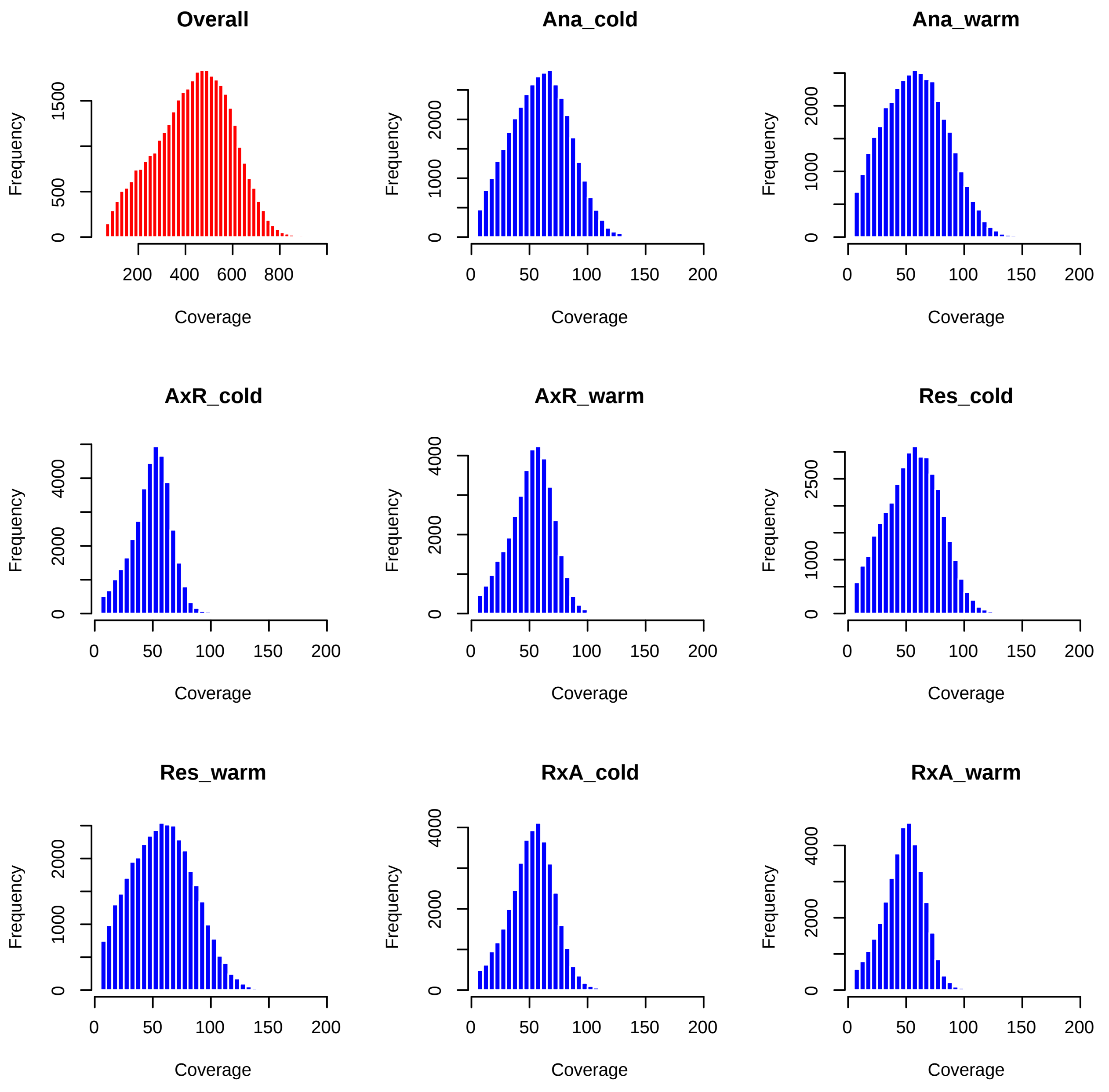

Fig. S2. Length distributions of genes and intergenic regions in the brown trout genome. Length is in the unit of bp and is logarithmized.

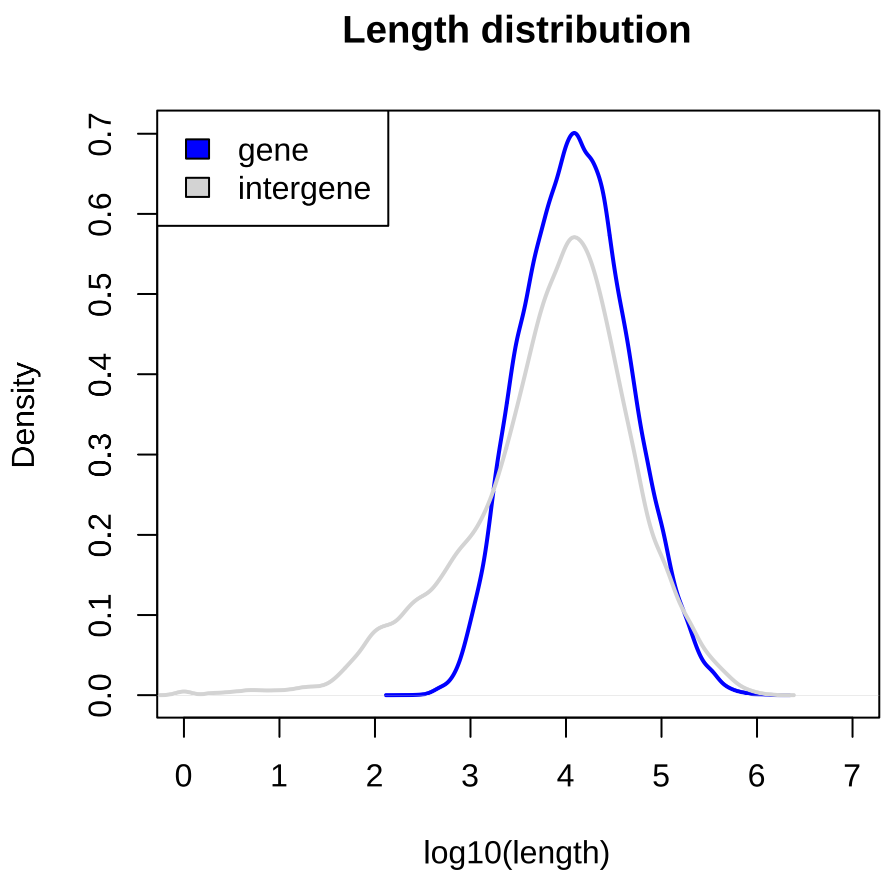

Fig. S3. Each plot shows the distribution of the neighboring distance among the CpGs with the methylation range indicated in the header. The number in the bracket shows the number of CpGs falling into this range. x-axis is log10(distance). y-axis is density. Yellow histogram shows the observed distribution. The grey histogram shows the expected distribution which was obtained by randomly sampling CpGs from the genome with the sample size equal to the number in the bracket.

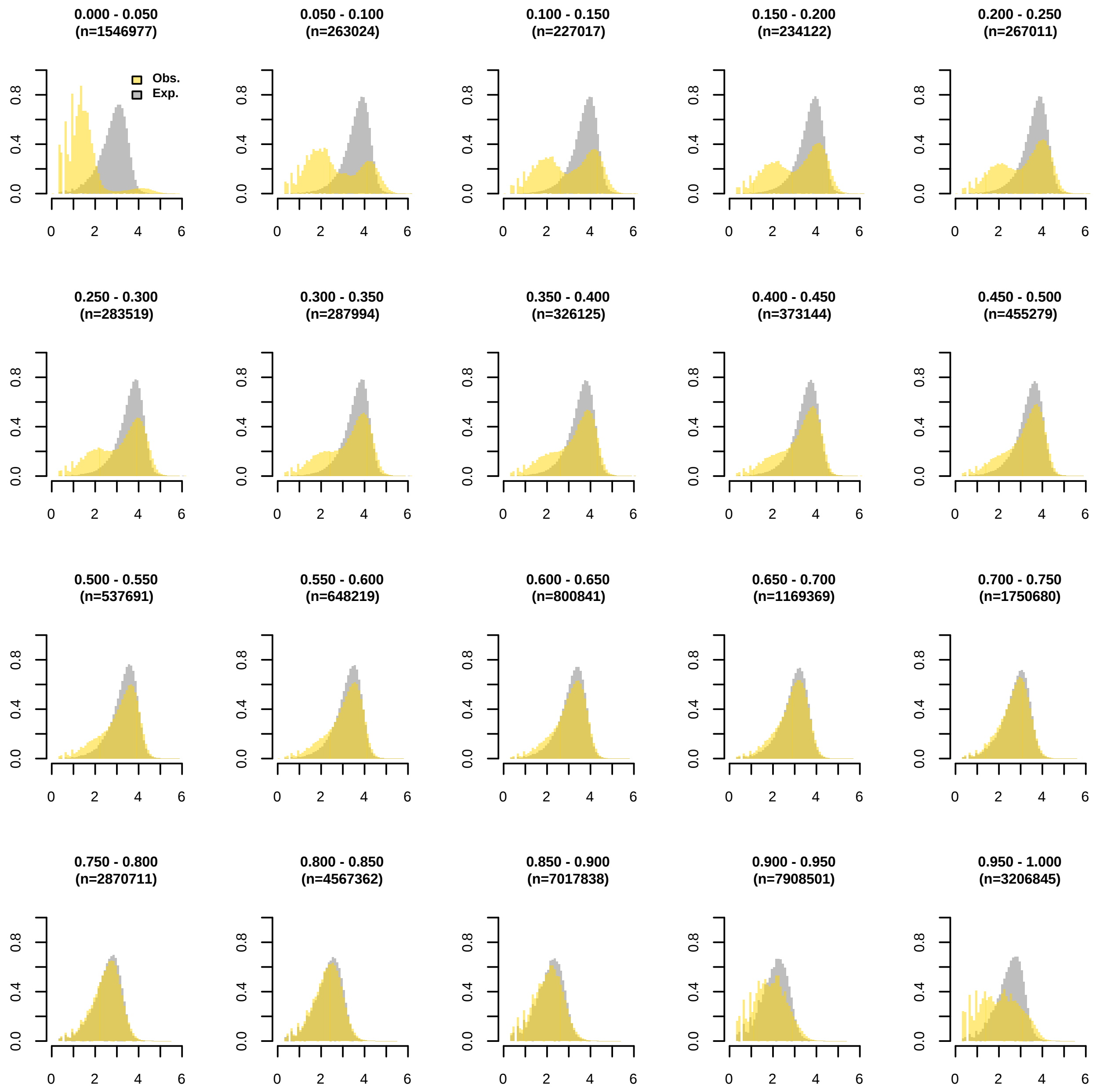

Fig. S4. Length distribution of the lowly-methylated regions (LMRs). See materials and methods for details.

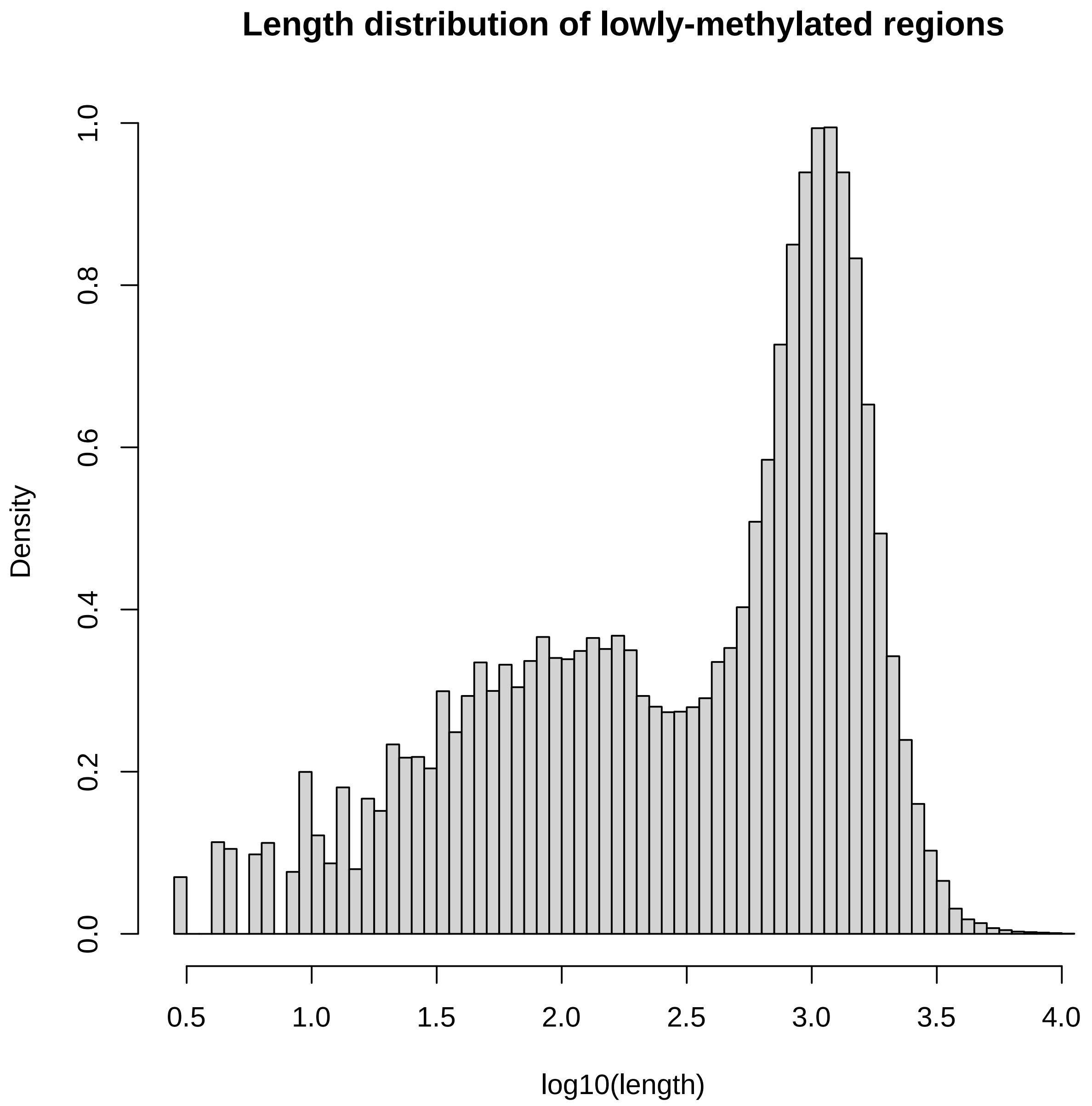

Supplement: Supplementary file 1 — Figures S1‐S4: ece372154‐sup‐0001‐FigureS1‐S4.pdf. [file ECE3-15-e72154-s002.pdf]
